# Fundamental Investigations into Metoprolol Tartrate Deposition on Orodispersible Films by Inkjet Printing for Individualised Drug Dosing

**DOI:** 10.3390/pharmaceutics13020247

**Published:** 2021-02-10

**Authors:** Olga Kiefer, Björn Fischer, Jörg Breitkreutz

**Affiliations:** 1 Institute of Pharmaceutics and Biopharmaceutics, Heinrich-Heine-University Düsseldorf, 40225 Düsseldorf, Germany; fischer@ramanservice.de (B.F.); joerg.breitkreutz@hhu.de (J.B.); 2 FISCHER GmbH, Raman Spectroscopic Services, 40667 Meerbusch, Germany

**Keywords:** pharmaceutical inkjet printing, orodispersible films, metoprolol tartrate, individualised medicine, paediatrics, Raman microscopy

## Abstract

Individualised medicine is continuously gaining attention in pharmaceutical research. New concepts and manufacturing technologies are required to realise this therapeutic approach. Off-label drugs used in paediatrics, such as metoprolol tartrate (MPT), are potential candidates for innovations in this context. Orodispersible films (ODFs) have been shown as an accepted alternative dosage form during the last years and inkjet printing is traded as seminal technology of precise deposition of active pharmaceutical ingredients (APIs). The objective of this study was to combine both technologies by developing imprinted ODFs based on hypromellose with therapeutically reasonable MPT single doses of 0.35 to 3.5 mg for paediatric use. After preselection, suitable ink compositions were analysed by confocal Raman microscopy regarding MPT distribution within the imprinted ODFs. Adjusted print settings, speed, print direction and angle, characterised the final ODF surface structure. The present investigations show that uniform dosages with acceptance values between 1 and 6 can be achieved. Nevertheless, changes in calibrated printed quantity due to nozzle aging have a significant effect on the final applied dose. At the lowest investigated quantity, the RSD was ±28% and at the highest, ±9%. This has to be considered for implementation of inkjet printing as a pharmaceutical production tool in the future.

## 1. Introduction

Beta(β)-blockers like metoprolol are essential for treatment of heart diseases in adults but also in premature infants, newborns and children [1]. Although safety and effectiveness in paediatric patients of all ages have not been proven yet, they are used off-label based on one’s total experiences [2,3]. In case of metoprolol, only the extended-release succinate salt received a Food and Drug Administration (FDA) approval, in 2007, for treatment of hypertension in children from 6 to 16 years [4,5]. The application of the commercially available preparations is not feasible because the dosages exceed the therapeutic range for premature infants and newborns. Furthermore, as a principle, metoprolol should be dosed gradually and under strict control. Up to now, capsules or liquid formulations are extemporaneously compounded in hospital or community pharmacies to fill these gaps [6,7]. For that purpose, metoprolol tartrate (MPT) is used as a rapidly dissolving salt. However, it is well-known that the dosing accuracy of capsules is prone to error, especially in case of low-dose formulations [8]. Application errors using dosing devices for liquid medicines are reported to be common [9]. To improve the access to safe oral cardiac drug therapy for children of all ages, advanced manufacturing techniques must be established.

A promising dosage form approach is provided by orodispersible films (ODFs). ODFs are oromucosal preparations consisting of water-soluble polymers that disintegrate in the mouth within a few seconds, enabling a flexible, safe and discrete intake. The most preferred manufacturing technology for small scale is the solvent-casting method [10]. ODFs have been discussed as a seminal dosage form for elderly with swallowing issues, non-cooperative patients as well as for children to avoid aspiration or choking [11,12]. Their commercial debut was as mouth freshener strips, Listerine^®^ PocketPaks, in 2000, followed by various applications containing active pharmaceutical ingredients (API) [13]. However, there is still only a limited number of existing medical products available. A further field of application, the individualised therapy, is simultaneously gaining popularity. In the concept of individualised medicines, ODFs may be produced in hospital or community pharmacies, tailored to the patient’s needs [10,14,15]. In case of paediatric patients, acceptance and even superiority in comparison to syrup have already been shown [16,17].

Various concepts have already been presented to realise this approach. Cutting devices were developed to allow flexible cutting of bulk ODF narrow rolls [18,19]. Electrospinning and three-dimensional (3D)-printing were used to manufacture in-situ individual tailored ODFs [20,21,22]. Finally, ODFs were impregnated with API-containing edible ink by flexographic [23], stencil [24] and inkjet printing [25,26,27,28,29], producing single-drug preparations or fixed-dose combinations. All mentioned technologies have their advantages and drawbacks, but they have in common that the fundamentals still need to be further investigated with regard to suitability for manufacturing dosage forms of pharmaceutical quality.

In this study, the focus is put on pharmaceutical inkjet printing as a potential technique for MPT deposition on drug-free ODFs. Inkjet printing is characterised by its precision, flexibility and contactless processing. Even small ink quantities can be reliably printed, selecting suitable print heads and settings [30], which is useful for printing expensive or potent drug substances [29,31]. Additionally, temperature-sensitive APIs can be processed in contrast to cast ODFs where solvents have to be removed by drying at high temperatures. However, recrystallisation issues at the nozzle plate and clogging by particles out of the processable range are challenges [32]. 

First studies have already been conducted on commonly prescribed low-dose APIs in paediatrics and their suitability for pharmaceutical inkjet printing on ODFs in the Netherlands [33]. However, one of the exclusion criterions for ODFs as a dosage form of first choice was the low off-label utilisation of the API. As a result, many APIs had to be omitted for further consideration. The frequency of APIs used may vary from country to country and hospital to hospital. Nevertheless, these APIs are applied in clinical practice and most likely require optimised routes of administration. For instance, a study carried out in 2016 at German hospital pharmacies showed that MPT was among the top 10 most commonly used drugs [7], whereas it does not appear in the study from the Netherlands. This highlights the core issue that most of the medicines used in children do not have a legal status and are therefore often not taken into account for dosage form innovations. Major steps to improve this situation have already been established with the introduction of the paediatric investigation plan (PIP) by the European Medicines Agency (EMA) and Food and Drug Administration (FDA) [34]. The β-blocker MPT is one of many APIs which could come into question for development of appropriate dosage forms [35]. The present fundamental investigations were intended to make a contribution to this. The distribution of MPT within the ODFs after inkjet printing, uniformity of dosage units and stability of calibrated contents were examined. These aspects are not yet sufficiently considered in the pharmaceutical field.

## 2. Materials and Methods

### 2.1. Preparation of Orodispersible Films

ODFs were produced on lab-equipment using the solvent-casting method: 15% (*w*/*w*) of hypromellose (HPMC, Pharmacoat^®^ 606, Shin-Etsu, Japan) as a film-forming agent and 3.5% (*w*/*w*) of glycerol 85% (Caeser & Loretz, Hilden, Germany) as a plasticiser were dissolved in deionised water under stirring. An automated film applicator (Coatmaster 510, Erichsen, Hemer, Germany) equipped with an electrically heated vacuum suction plate was used to cast the polymer solution on the process liner consisting of a polyamide/polyester mixture (Mediflex^®^ XM AMWL 45/105, Amcor Flexibles, Gent, Belgium). The adjustable coating knife (Multicator 411/220 mm, Erichsen, Hemer, Germany) was set to a gap height of 500 µm at a drawing speed of 6 mm/s. Subsequently, the drug-free film sheets were dried at 50 °C and cut to a size of 22 × 29 cm, matching the substrate table dimensions of the inkjet printer.

### 2.2. Ink Formulations

For preparation of the inks, metoprolol tartrate (MPT, Microsin, Bucharest, Romania) was weighed and dissolved in the appropriate volume of the water under stirring at ambient temperature. In case of drug-free inks, this step was skipped. Poloxamer 407 (P407, Lutrol^®^ F 127, BASF, Ludwigshafen, Germany), hypromellose (HPMC, Pharmacoat^®^ 615, Shin-Etsu Chemical, Tokyo, Japan) and glycerol 85% (Caesar & Loretz, Hilden, Germany) were added in various combinations, modifying the fluid’s properties (Table 1). 

#### 2.2.1. Dynamic Viscosity

To determine the dynamic viscosity, the rotational rheometer Kinexus pro (Malvern Instruments, Worcestershire, UK) equipped with a cone (1°/Ø 60 mm; CP1/60 SR2482 SS) and plate geometry (Ø 65 mm; PL65 S0520 SS) was used. The measurements were performed at 30 °C and a shear rate of 1000 s^−1^. Each sample was measured in triplicate. Per measurement, 60 values were recorded, and arithmetic means and standard deviations (SD) were calculated.

#### 2.2.2. Surface Tension

Surface tensions of the ink solutions were determined by the automatic force tensiometer K100 (Krüss, Hamburg, Germany) using the Wilhelmy plate method at 30 °C. The samples were measured in triplicates. Per measurement run, 10 values were recorded, and arithmetic means and standard deviations were calculated.

### 2.3. Inkjet Printing

Inkjet printing was performed using the desktop inkjet system PixDro LP50 (Meyer Burger, Eindhoven, The Netherlands) equipped with the piezo-driven drop-on-demand print head Spectra SE-128 AA (SP, Fujifilm Dimatix, Santa Clara, CA, USA). The temperature of SP was set to 30 °C and the temperature of the substrate table to 25 °C, ensuring controlled conditions. Pulse voltage was adjusted to 100 V (90%) and the negative pressure applied to the ink supply of −27 mbar related to normal pressure. The distance of print head to the substrate was 1 mm. Printing was conducted by only one nozzle of varying number. A rectangular template with a defined area was created by editing software, Photoshop CS2 (Adobe, San José, CA, USA).

The integrated drop view system of the inkjet printer PixDro LP50 equipped with the software tool Advanced Drop Analysis (ADA, version v2.4.2) was used to investigate the printability of inks and optimise the waveform during preliminary studies.

#### 2.3.1. Printing Motion

To investigate the influence of printing settings on surface structure of ODFs, print speed as well as direction and angle were varied (Table 2). For that purpose, an area of 1 inch^2^ (=2.54 × 2.54 cm^2^) was printed, adjusting the print resolution to 500 dots per inch (dpi). These dimensions were selected to simplify the calculation of number of drops per area or line, as the resolution is measured in dots per inch (dpi).

**Figure 1:**
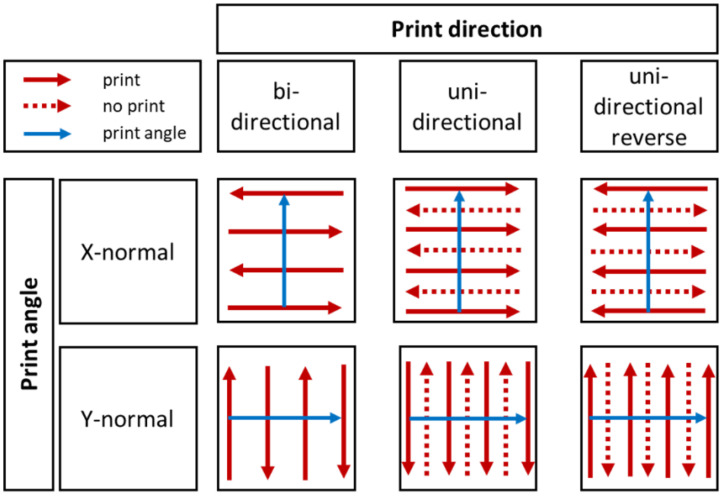
Illustration of used combinations of print direction (**red**) and angle (**blue**), modified according to the PixDro LP50 manual.

In this context, print speed is defined as speed of the substrate table below the print head. Print angle describes the coordinate direction of the print head movement. Finally, print direction determines the swaths printed by the print head (Figure 1).

#### 2.3.2. Printing Resolution

Square samples were printed at resolutions from 250 to 2000 dpi in equidistant steps of 250 dpi in randomised order. The print speed of the substrate table was calculated (Equation (1)) and adjusted between 25 and 200 mm/s to keep a constant jetting frequency of 2 kHz.

v = (25.4 × QF × f)/R_p_ (1)

v: printing speed (mm/s)

f: frequency (Hz)

QF: quality factor, number of nozzles used to print one line in in-scan direction

Rp: printing resolution (dpi)

The resulting MPT content regression curve was used to calculate the required resolution for the target dosages. To examine the inter-nozzle variation regarding printed quantities, one-way analysis of variance (ANOVA) with subsequent post hoc Tukey’s test was performed at significance level of α = 0.05.

### 2.4. Confocal Raman Microscopy

Microscopic Raman investigations were performed using the confocal Raman microscope alpha300 R (WITec, Ulm, Germany). A fibre-coupled single-mode laser with 532 nm excitation wavelength was used. The laser power on the samples was set to 20–23 mW. A Zeiss EC Epiplan-Neofluar HD 100×/0.9 NA and a Zeiss EC Epiplan-Neofluar HD 20×/0.5 were selected as microscope objectives. WITec UHTS 300 was used as a spectrometer in combination with an Andor iDus Deep Depletion charge-coupled device (CCD) detector, which was cooled to −60 °C. The Raman scattered light was spectrally dispersed at a reflection grating with 600 lines/mm. An average spectral resolution of about 3.8 cm^−1^/pixel was achieved. The evaluation of the measurement data and the creation of the Raman images was performed using the software FIVE (version 5.2.4.81, WITec, Ulm, Germany), including a cosmic ray removal and background subtraction by the implemented shape function. Unless otherwise stated, the spectra were normalised related to the HPMC peak at 1370 cm^−1^, correcting arisen scattering losses due to increasing penetration depth.

Furthermore, the Raman microscope was used in dark field mode to investigate the surface structure of printed ODFs at different print settings (Section 2.3.1). Using the microscope objective Zeiss EC Epiplan-Neofluar HD 20×/0.5, stitching images were recorded and combined to an overview of 2000 × 2000 µm (M1–M3) or 2000 × 600 µm (M4–M12).

### 2.5. Assay

MPT content of ODFs was analysed using high-performance liquid chromatography (HPLC). The analytical HPLC system Agilent 1260 Infinity (Agilent Technologies, Santa Clara, CA, USA) consisted of a binary pump G1312B, autosampler G1329B, temperature-controlled column compartment G1316A, degasser G4225A and diode array detector (DAD) G4212B. The C18-column ODS Hypersil^TM^ with the dimensions 150 × 4 mm and particle size of 3 µm (Thermo Fisher Scientific, Waltham, MA, USA) was used. 10 µL of sample was injected and analysed at a wavelength of 221 nm. A mixture of phosphate buffer (4.6 mM, pH 3) and acetonitrile (15:85) was used as eluent for the isocratic method. The flow rate was set to 2.0 mL/min at a column temperature of 25 °C.

### 2.6. Uniformity of Dosage Units

The content uniformity (CU) was assessed according to Ph. Eur. monograph 2.9.40. The content of ten single doses was determined by HPLC analysis. The mean of individual contents, $\bar{X}$, expressed as percentage of the label claim, was calculated and inserted into Equation (2) in order to determine the acceptance value (AV):

(2) $$\mathrm{AV}=|M-\bar{X}|+k\;s$$

The reference value, M, was chosen depending on $\bar{X}$ (case 1: T = 100.0%). The acceptability constant, k, is 2.4 (*n* = 10) and s is defined as sample standard deviation. The maximum allowed AV is 15.0 for test level 1 (L1). If the AV exceeded 15.0, no further 20 single doses as specified in Ph. Eur. 2.9.40 at L2 were tested.

### 2.7. Mechanical Properties

The puncture strength and elongation at break of the imprinted ODFs were investigated by the texture analyser TA.XT*plus* (Stable Micro Systems, Godalming, UK), equipped with a cylindrical flat-faced aluminium probe of 5.0 mm diameter [36]. The samples were clamped between two plates with a circular recess of 10.0 mm diameter. The probe was moved perpendicular to the sample at a speed of 1.0 mm/s. After a trigger force of 1 N was reached, a distance of 5.0 mm was covered. The results were compared with unprinted HPMC substrate.

## 3. Results

### 3.1. Ink Formulation Development

As MPT is very good soluble in water according to Ph. Eur., distilled water was chosen as a carrier fluid for the ink. The advantage of water for drug printing is that it is an uncritical solvent regarding toxicity. A potential hazard is bacterial or fungi growth during storage. However, preservatives should be avoided for use as ink for paediatric population, especially neonates [37]. Therefore, the formulations are intended for processing within 24 h. An addition of preservatives could be considered for older children only. 

A surfactant has to be used because water shows a surface tension of 71.2 mN/m at 30 °C, which is too high for stable inkjet printing [38]. Poloxamer 407 (P407) was selected at a concentration of 0.1% (*w*/*w*) due to its non-ionic character so that less interactions with ionic APIs could occur. As jetted fluids can be accompanied by secondary droplets, so-called satellites, 1% HPMC was added to modulate the ink viscosity and stabilise the drop ligament [39]. Additionally, 10% glycerol was used as a viscosity enhancer but mainly as a humectant to prevent too-fast water evaporation. 

#### 3.1.1. Physicochemical Properties

In order to investigate the influence of the individual components on the ink properties, drug-free solutions (I1–I4) were analysed in addition to MPT-containing formulations (I5–I7). Pure water (R) served as a reference.

Regarding the key properties of the prepared fluids, it is noticeable that surface tensions of the inks I1–I7 are quite similar at about 40 mN/m (Table 3) and mainly depend on the use of surfactant P407. Dynamic viscosity, on the contrary, is clearly influenced by the total ink composition. The addition of MPT results in a greater change of ink viscosity if HPMC is already present in the solution. This can be described by the solvation of dissociated MPT salt. Fewer water molecules are available for the hydration of HPMC chains so that the dynamic viscosity increases. Glycerol 85% as a liquid with dynamic viscosity of 61 ± 1.5 mPa*s at 30 °C leads to a further increase. Finally, the fluid density is affected by the solid content as well as by the amount of glycerol. In summary, all components have a certain impact on the physicochemical ink characteristics, which makes a systematic ink development more complex.

#### 3.1.2. MPT distribution within the ODFs

**Figure 2:**
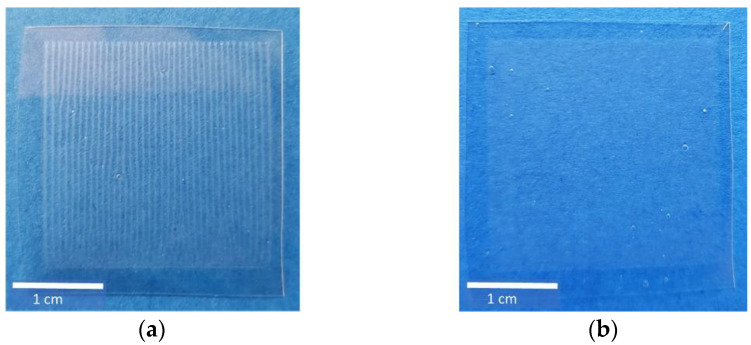
Ink formulations I5 (**a**) and I7 (**b**), printed on HPMC-ODFs using a resolution of 500 dpi, resulting in 500 × 500 drops on an area of 1 inch^2^ (≈6.45 cm^2^).

With the help of a drop analysis method, I5 and I7 were identified as the most promising formulations in preliminary studies. To figure out the most suitable formulation on a discrimination basis, the distribution of MPT after printing both inks was investigated. Already, visually, a difference between I5 and I7 could be detected (Figure 2). HMPC-ODFs imprinted by I5 showed a regular line pattern, whereas the ones imprinted by I7 showed a blurred structure. Counting the lines of I5-ODF, however, the theoretical number of 500 deriving from the set resolution was not reached, and only 38 lines could be recovered. This could indicate that the drop diameters are larger than the swath width, but it still does not explain why I5 and I7 resulted in different print patterns.

**Figure 3:**
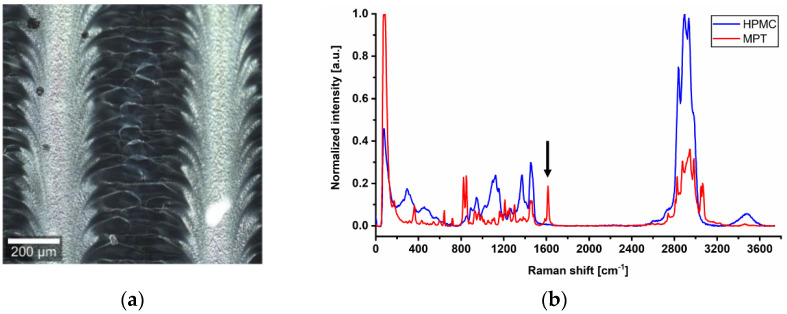
(**a**) Dark field image of ODF imprinted by I5. (**b**) Raman spectra of pure substances HPMC and MPT with a characteristic peak at 1615 cm^−1^ and 10 s exposure time, normalisation to the intensity of respective maximum peak in arbitrary unit (a.u.).

To examine the differences in more detail, confocal Raman microscopy was used. In the dark field, the previously mentioned line pattern of I5-ODF turned out to be a feather-like structure (Figure 3a). This strengthened the hypothesis of an unequal distribution of MPT. The explanation could be found in the partial line-by-line dissolving of the film surface by the water-based ink during printing, but it does not reflect the mismatch of visible line number and set resolution. 

For Raman mapping, a characteristic peak had to be found for MPT which is not influenced by the second main component HPMC. After analysis of the spectra of pure substances, the MPT peak at 1615 cm^−1^ (Figure 3b) was chosen.

**Figure 4:**
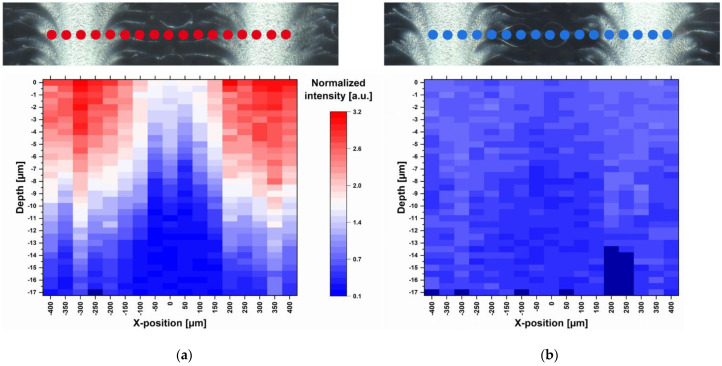
Dark field images and corresponding heatmaps of single-point depth scans (17 positions) at 1615 cm^−1^ recorded with spatial resolution of 0.5 µm and 10.0 s exposure time (**a**) one day after printing and (**b**) 2.5 months after printing, normalisation related to the HPMC peak at 1370 cm^−1^; red: high intensity, blue: low intensity, dark blue: no data available.

Raman measurements of a freshly manufactured sample stored for one day in a desiccator at 18 °C were performed. 17 points were selected along a line of 800 µm so that the light and dark feather-like structures could be captured (Figure 4). Small-stepped scans were performed at each point with a spatial resolution of 0.5 µm and 10.0 s exposure time along a depth of 20 µm. Since the measurements could not always start exactly at the same distance from the ODF surface, the first measurable Raman intensity maximum was defined as surface (depth = 0 µm). In total, 577 Raman spectra were recorded along 17 µm depths, which are about half the thickness of the film. With increasing depth, the Raman intensity decreased due to the decreasing MPT concentration and not due to the increasing deflection of the laser. This was confirmed by a two-point measurement of the film back side, which resulted in no presence of MPT. Furthermore, an intensity profile corresponding to the surface structures becomes obvious. There is a higher MPT concentration in the light areas of the dark field image than in the darker areas. This actually means that the regular deposited ink drops coalesce on the ODF before drying and that the drops have not been deposited regularly at all. This topic is discussed in more detail in the following section.

After short storage of one day at low relative humidity (RH) and temperature (15–30% RH, 18 °C) directly after printing, the diffusion of MPT was limited to the upper half of the ODF (Figure 4a). After a further storage of the sample in polyethylene-sachet at room conditions with higher temperature and relative humidity (40–60% RH, 22–25 °C) for 2.5 months, the diffusion took place throughout the whole film thickness. This led to a homogenous MPT distribution but smaller Raman intensities due to lower concentration per film volume (Figure 4b).

**Figure 5:**
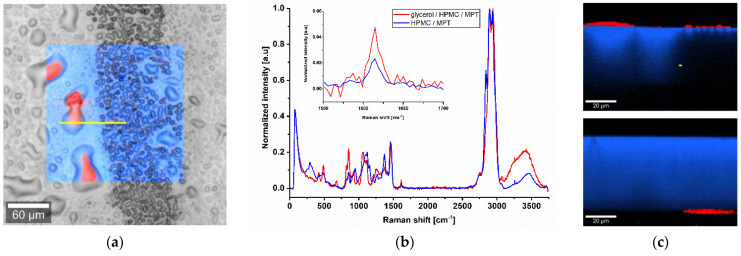
Raman microscope images of ODF imprinted by I7. (**a**) Surface scan with an area of 200 × 200 µm, spatial resolution of 1 µm and 0.05 s exposure time. (**b**) Average normalised Raman spectra of the surface scan with magnified characteristic MPT peak at 1615 cm^−1^. (**c**) Depth scan of the cross-section from top and bottom side of the ODF with an area of 100 × 70 µm along the yellow line in (**a**), spatial resolution of 1 µm and 0.2 s exposure time. Red: mixture of glycerol, HPMC and MPT; blue: mixture of HPMC and MPT.

In contrast, I7 ink stayed partially on the surface of the ODF and accumulated the dissolved MPT (Figure 5a). The ink could be easily wiped off by touching or packaging the ODF. The reason is that glycerol did not evaporate within an appropriate process time and was not completely incorporated into the film matrix. Porous or functionalised substrates could be an alternative to solve this issue [25,41]. A depth scan (Figure 5c) was performed along the yellow line (Figure 5a). The printed side was placed upwards. By analysing the Raman spectra (Figure 5b), a qualitative assignment of the components could occur. The red marked area displays a mixture of glycerol, HPMC and MPT, and the blue marked area a mixture of HPMC and MPT. For an accurate quantitative statement, a further calibration step should be carried out. It can be noticed that glycerol drops containing MPT are located on the ODF surface. Since the drops deflected the Raman signal, there are black unassigned spaces. To verify that the whole film consists mainly of HPMC, the film sample was turned over so that the printed side was facing down. As it is challenging to find exactly the same spot again, the investigated area was slightly shifted. Nevertheless, a drop of glycerol is visible on the bottom side. The black area is replaced by a continuous blue one because of the smooth surface, and only a small air bubble in the film matrix can be anticipated. By performing Raman mapping, it was feasible to determine the localisation of MPT after printing of two potential ink formulations and draw further conclusions based on this. 

In case of ODFs which disintegrate rapidly and are being swallowed with saliva flow, the distribution of API and ink excipients within the film matrix after inkjet printing and during storage primarily serves to differentiate between suitable and unsuitable ink formulations. Regarding further film application like mucoadhesive buccal films, however, the uncontrolled API diffusion could be crucial if unidirectional dissolution has to be assured. There could be a risk of undesirable co-dissolving of the directly subsequent protecting backing layer during printing of high ink quantities onto the mucoadhesive film layer. Confocal Raman microscopy offers a helpful tool to investigate and evaluate these phenomena on a small scale.

### 3.2. Surface Structure of Printed ODFs

Further investigations were carried out to determine the cause of the patterns on the ODF surface after inkjet printing. After varying the print settings as described in Section 2.3.1, the samples were viewed under the confocal Raman microscope in the dark field. This enabled a defined visualisation of the surface structures.

**Figure 6:**
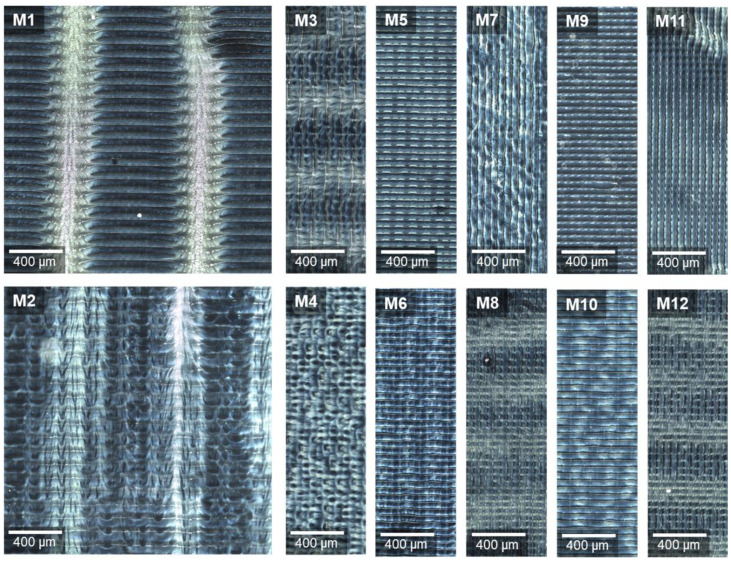
Dark field images of ODFs imprinted at varying print settings (defined in Table 2), stitching images of an area 2000 × 2000 µm (M1–M2, M3 cut) and 2000 × 600 µm (M4–M12), rotated by 90° for observation under the microscope.

Figure 6 displays the generated images. All have the regular stripe pattern in common. X-normal print angle resulted in 40 parallel, and Y-normal print angle in 12 perpendicular stripes, referring to the front edge of the substrate table. Relating this to the whole printed area of 2.54 × 2.54 cm^2^, then, in both cases, a stripe width of exactly 50 µm is obtained. This means that a width of 2.54 cm includes approximately 500 stripes, which corresponds to the set resolution of 500 dpi and with that to the number of printed swaths.

Comparing the prepared samples at low and high speed, it can be noticed that the 100 mm/s (Figure 6, even numbers) led to a more diffuse pattern than 50 mm/s (Figure 6, odd numbers). This may be caused by more turbulent air flows in the gap between the moving print head and substrate table at higher speed. The feather-like pattern appears clearly only with bidirectional inkjet printing (M1, M2). In M3, M8 and M12, only blurred light areas perpendicular to the printed swaths are visible. 

The preceding experiments showed that the light areas contain higher MPT concentration directly after finishing the printing process. The jet from a nozzle usually consists of main drops and satellites. The latter are undesirable but can often only be reduced and not completely avoided. Due to their small volume, and with that mass, secondary droplets in particular can be deflected by eddy flows [42,43]. Since HPMC was used as a film-former for ODFs, the surface was dissolved by the water-based ink drops which coalesce to stripes. Each dried partially before an adjacent swath was printed. Depending on the print motion settings, the drops flew at different angles on the substrate, creating feather-like three-dimensional surface structures. The additional ink deposition at the areas already printed with main drops led to a structural change and higher MPT content. The explanation of the observed repetitive regularity, however, remains open.

### 3.3. Paediatric Individual ODFs

#### 3.3.1. Dosage Precision by Printing Resolution

The API dosing can be controlled by means of ink API concentration, number of layers, printed area and resolution. All adjustments have individual advantages and disadvantages. The ink concentration is limited by the API solubility in case of solutions. As long as the API remains dissolved, different concentrated inks could serve as bulk product. The number of layers is not freely selectable due to limited mechanical stability of the film substrates and especially the prolongation of process time, but printing several layers allows an easy adjustment of the dose without an ink exchange and cleaning step. By pre-setting different template areas, the physiological facts of target patients have to be considered. The oral cavities of newborns, children and adults differ significantly in surface area [44]. As the ODFs have to be cut with excess to the sides of the printed area to ensure no loss of API, the final sizes have to be planned. Using different printing resolutions enables the increase of API dosing without exchanging the ink and expanding the production time if selecting a high-resolution inkjet print head. 

In the present study, the printing resolution was chosen as a parameter for dosage control. For the production of paediatric tailored MPT dosages, downscaling of printed area to an appropriate child-friendly size of 1 cm^2^ was performed by choosing the most suitable ink composition, I7. Although MPT is administered only off-label to children, there are some recommendations in the literature. For treatment of hypertension, initial doses of 1–2 mg/kg body weight/day distributed over two single doses are reported. Maximum doses are indicated as 6–8 mg/kg body weight/day up to a maximum of 200 mg total dose per day. Twice daily administration of 0.1–0.2 mg/kg body weight is suggested for treatment of heart failure. The dose can be doubled every two weeks in stable patients until the target dose of 1–2.5 mg/kg body weight is reached [45,46]. 

**Figure 7:**
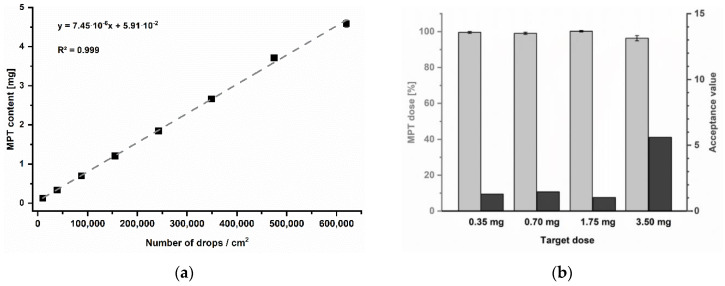
(**a**) Calibration series of printing resolutions, mean ± SD (*n* = 3). (**b**) Resulting MPT dose as percentage of label claim of printed units, mean ± SD (*n* = 10) and corresponding acceptance values.

For the present trials, a newborn of 3.5 kg was exemplary chosen as a target patient for simulating a very low-dose regime. Regarding the information mentioned before, single dosages of 0.35 to 0.7 mg and 1.75 to 3.5 mg are indicated for initial heart failure treatment. The print resolution was selected as a variable parameter to achieve the target dosage strengths. To systematically determine which resolutions might be required, a calibration series was performed in the range of 250 to 2000 dpi (Figure 7a). Since print resolution is typically specified in drops per inches (dpi), for practical reasons, the resulting MPT content was instead related to the number of drops in a printed area of 1 cm^2^ (Table 4). Furthermore, the print speed was adjusted according to resolution to keep a constant frequency of 2 kHz. Thus, a potential effect of frequency on the printed quantity could be excluded.

The resulting MPT contents could be described by a linear mathematical function with a coefficient of determination of R^2^ = 0.999. The linear equation was used to calculate the required resolutions to accomplish the recommended initial single MPT dosages for 3.5 kg newborns: 0.35 and 0.7 mg for heart failure as well as by 1.75 or 3.5 mg per unit for treatment of hypertension. All batches achieved the target doses, showing low AV for the pharmacopoeial specified limit of 15 at L1 (Figure 7b).

**Figure 8:**
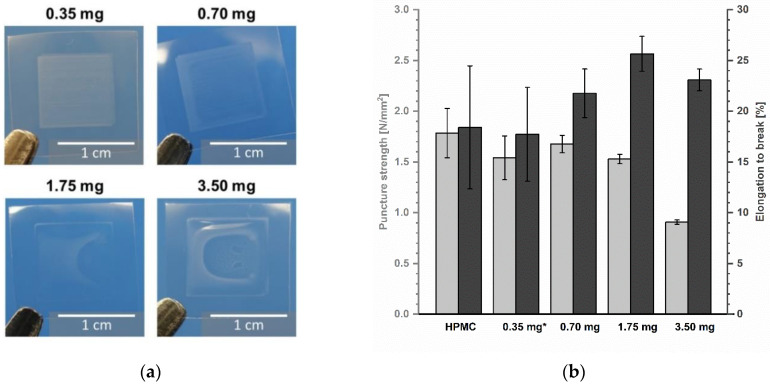
(**a**) Images of ODFs with printed area of 1 cm^2^. (**b**) Mechanical properties of ODFs measured by texture analyser, mean ± (SD) (*n* = 3, * *n* = 2).

The mechanical properties of printed ODFs were analysed in comparison to non-printed drug-free ODFs made from HPMC to determine the influence of the deposited ink. Looking at Figure 8b, it is obvious that the inkjet printing has a minor influence on the resistance of the ODFs, although macroscopically, a change of shape is visible (Figure 8a). On the contrary, the film matrix seems to become more even by partial redissolving and drying so that the standard deviations of puncture strength as well as elongation to break vary less with increasing ink amount. The highest ink application led to the lowest puncture strength due to physical deformation of the film during printing. Thereby, the deposited water by the ink served as an additional plasticizer and caused more elastic films and increasing elongation at break. To gain further information about the physical properties of imprinted ODFs, the influence on disintegration behaviour could be investigated. Since ODFs are by definition a rapidly disintegrating dosage form, it is assumed that the disintegrating time will be further accelerated by impregnation with aqueous solutions.

#### 3.3.2. Content Variation

**Figure 9:**
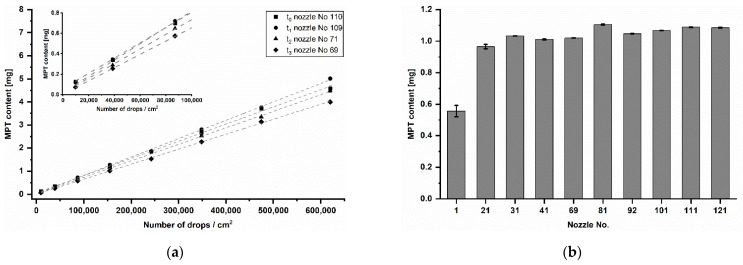
(**a**) Calibration series of printing resolution at different time points (t_0_–t_3_) distributed over five months, mean ± SD (*n* = 3). (**b**) MPT content of samples printed at 1000 dpi with ten different nozzles on the same day in randomised order, mean ± SD (*n* = 3).

Performing the calibration step before each inkjet printing process in further studies, it was noticed that the MPT dose varied over time (Figure 9a). The resolution of 250 dpi led to quantities between 73 and 127 µg and 2000 dpi to quantities between 4 and 5 mg. However, the coefficient of determination was at each time point R^2^ ≥ 0.999. Since different nozzle numbers (No.) were used for inkjet printing, it was assumed that there is an inter-nozzle variation in terms of drop volume. To verify this, samples were printed with one of ten nozzles distributed over the entire print head width. The resolution of 1000 dpi was chosen as it is almost in the centre of the analysed resolution range. The production was done in one day. The resulted mean MPT contents are displayed in Figure 9b. According to the performed Tukey’s test, only nozzle No. 1 differed significantly from other used nozzles. The remaining mean values showed non-significant differences around 1 mg.

If the means of the respective resolutions printed at different days over five months (Figure 9a) are compared by the Tukey’s test, it turns out that almost all are significantly different from each other. Only three mean differences of t_0_ and t_1_ of a total of 45 combinations are non-significant. Looking at the values in the course of the time from t_0_ to t_3_, a decreasing MPT content in total is apparent. For this reason, a general inter-nozzle variation is considered less relevant than the nozzle aging and its potential blockage over time. For the pharmaceutical inkjet printing, it means that a calibration series in the target range before each printing process is crucial, especially after a long manufacturing break. Each time, the resolutions for target API strengths have to be recalculated using a recent calibration series. If this is assured, then the inkjet printing is not only very precise but also accurate in dosing.

## 4. Conclusions

A series of formulations were investigated with the aim of developing printable drug-loaded inks for manufacturing of individual-dose ODFs with metoprolol tartrate. The target patient group, paediatric patients of different ages, require dosage accuracy in the production of low-dose drugs. It was found that glycerol is not suitable as an excipient with the function to prevent drying at the nozzles for inks printed on ODFs based only on HPMC. Due to a lack of penetration into the substrate and evaporation, an API loss-free handling and packaging cannot be ensured. A more absorbent film substrate has to be developed to overcome this issue. In this context, confocal Raman microscopy was proven to be a useful tool, providing three-dimensional chemical mapping. Overall, precise deposition of uniform MPT doses was feasible. The change of calibrated contents during a production period of five months shows, however, that further experiments are required to optimise the pharmaceutical inkjet printing process. A feather-like surface structure of imprinted ODFs was discovered. Further investigations at different print settings indicate different aerodynamics underneath the print head as the main reason for this underived property.

## Figures and Tables

**Table 1:** Composition of investigated ink formulations (*w*/*w* %).

Ink Formulation	P407 (%)	Glycerol 85% (%)	HPMC 615 (%)	MPT (%)	Water (%)
I1	0.1	-	-	-	99.9
I2	0.1	10	-	-	89.9
I3	0.1	-	1	-	98.9
I4	0.1	10	1	-	88.9
I5	0.1	-	-	10	88.8
I6	0.1	-	1	10	87.8
I7	0.1	10	1	10	78.9

**Table 2:** Overview of print settings.

Sample	Print Speed (mm/s)		Print Angle		Print Direction		
	50	100	X- Normal	Y- Normal	Bi- Directional	Uni- Directional	Uni- Reverse
M1	X		X		X		
M2		X	X		X		
M3	X			X	X		
M4		X		X	X		
M5	X		X			X	
M6		X	X			X	
M7	X			X		X	
M8		X		X		X	
M9	X		X				X
M10		X	X				X
M11	X			X			X
M12		X		X			X

**Table 3:** Results of physicochemical investigations at 30 °C, mean ± standard deviation (SD) (*n* = 3).

Ink Formulation	Dynamic Viscosity at 1000 s^−1^ (mPa*s)	Surface Tension (mN/m)	Density (kg/m^3^)
R	0.8 ± 0.0	70.1 ± 0.6	995.7 [40]
I1	0.8 ± 0.0	39.8 ± 0.3	998.2 ± 3.4
I2	1.0 ± 0.0	39.7 ± 0.4	1019.4 ± 1.6
I3	3.1 ± 0.1	40.3 ± 0.6	1000.4 ± 2.1
I4	4.1 ± 0.1	39.7 ± 0.5	1019.7 ± 3.4
I5	1.2 ± 0.0	38.3 ± 0.5	1013.7 ± 2.5
I6	5.2 ± 0.1	38.7 ± 0.7	1007.6 ± 12.1
I7	6.2 ± 0.1	39.4 ± 0.4	1040.3 ± 2.2

**Table 4:** Used resolutions for calibration and corresponding calculated required print speed and number of drops related to an imprinted area of 1 cm^2^, dpi = drops per inches.

Print Resolution (dpi)	Print Speed (mm/s)	Number of Drops (1/cm^2^)
250	200	9688
500	102	38,750
750	68	87,188
1000	51	155,000
1250	41	242,188
1500	34	348,751
1750	29	474,688
2000	25	620,001

## Data Availability

The data presented in this study are available in “Fundamental investigations into metoprolol tartrate deposition on orodispersible films by inkjet printing for individualised drug dosing”.
